# Relationship between adductor pollicis muscle thickness and subjective global assessment in a cardiac intensive care unit

**DOI:** 10.5935/0103-507X.20150062

**Published:** 2015

**Authors:** Fernanda Pickrodt Karst, Renata Monteiro Vieira, Sandra Barbiero

**Affiliations:** 1 Instituto de Cardiologia, Fundação Universitária de Cardiologia do Rio Grande do Sul - Porto Alegre (RS), Brazil.

**Keywords:** Muscles, Thumb, Nutritional assessment, Risk measurement

## Abstract

**Objective:**

To verify the relationship between the adductor pollicis muscle thickness test and the subjective global assessment and to correlate it with other anthropometric methods.

**Methods:**

This observational cross-sectional study was conducted in the intensive care unit of a cardiology hospital in the state of Rio Grande do Sul, Brazil. The hospitalized patients underwent subjective global assessment and adductor pollicis muscle thickness tests on both hands, along with measurement of the right calf circumference. Laboratory parameters, length of stay, vital signs and electronic medical record data and tests were all collected.

**Results:**

The study population included 83 patients, of whom 62% were men. The average age was 68.6 ± 12.5 years. The most common reason for hospitalization was acute myocardial infarction (34.9%), and the most common pathology was systolic blood pressure (63.9%), followed by *diabetes mellitus* (28.9%). According to subjective global assessment classifications, 62.7% of patients presented no nutritional risk, 20.5% were moderately malnourished and 16.9% were severely malnourished. Women had a higher nutritional risk, according to both the subjective global assessment and the adductor pollicis muscle thickness test, the cutoff for which was < 6.5mm (54.8%; p = 0.001). The pathology presenting the greatest nutritional risk was congestive heart failure (p = 0.001). Evaluation of the receiver operating characteristic (ROC) curve between adductor pollicis muscle thickness and subjective global assessment showed the accuracy of the former, with an area of 0.822.

**Conclusion:**

Adductor pollicis muscle thickness proved to be a good method for evaluating nutritional risk.

## Abstract (pt)

**Objetivo:**

Verificar a relação do teste de espessura do músculo adutor do polegar com avaliação subjetiva global e correlacioná-lo a outros métodos antropométricos.

**Métodos:**

Estudo transversal observacional realizado em unidade de terapia intensiva de um hospital de cardiologia no Estado do Rio Grande do Sul. Os pacientes internados foram submetidos à avaliação subjetiva global, da espessura do músculo adutor do polegar em ambas as mãos e da circunferência da panturrilha da perna direita. Foram coletados parâmetros laboratoriais, tempo de internação, sinais vitais, dados e exames do prontuário eletrônico.

**Resultados:**

População composta de 83 pacientes, sendo 62% homens, com idade de 68,6 ± 12,5 anos. O motivo de internação mais frequente foi infarto agudo miocárdio (34,9%), e a patologia mais comum foi a hipertensão arterial sistólica (63,9%) seguida de *diabetes mellitus* (28,9%). Conforme a classificação da avaliação subjetiva global, 62,7% dos pacientes apresentaram-se sem risco nutricional, 20,5% moderadamente desnutridos e 16,9% gravemente desnutridos. As mulheres apresentaram maior risco nutricional, tanto pela avaliação subjetiva global quanto pela espessura do músculo adutor do polegar, que foi < 6,5mm (54,8%; p = 0,001). A patologia com maior risco nutricional foi a insuficiência cardíaca congestiva (p = 0,001). A avaliação da curva ROC entre espessura do músculo adutor do polegar e avaliação subjetiva global teve acuidade, com área de 0,822.

**Conclusão:**

A espessura do músculo adutor do polegar demonstrou ser um bom método de avaliação de risco nutricional.

## INTRODUCTION

In Brazil, the World Health Organization (WHO) estimates that in 2008, about 17.3 million people died from cardiovascular diseases, among which 7.3 million due to coronary heart disease.^(1)^

Patients with cardiovascular disease often require treatment in intensive care units (ICU), and malnutrition then becomes a common problem.^(2)^ The state of malnutrition is usually diagnosed using tools such as the subjective global assessment (SGA) and the mini nutritional assessment (MNA^®^).^(3)^ A literature review for the period 1998 to 2012 showed that the prevalence of malnutrition in hospitalized seniors ranges from 2% to 80%. This diversity is due to several factors, including the heterogeneity of the population.^(4)^

The SGA is currently widely used and is considered to be the gold standard for subjective evaluation, as it includes questions relating to weight loss, eating habits, gastrointestinal symptoms, functional capacity, stress of the base disease and physical examination.^(5)^ However, a survey of 526 patients in an institution specializing in cardiology evaluated the use of different nutritional assessment tools and suggested that the use of a single tool of this nature is insufficient for a correct and reliable diagnosis of malnutrition in cardiac patients.^(6)^

An evaluation technique that has been used to estimate muscle loss and hence malnutrition is the measurement of adductor pollicis muscle thickness (APMT).^(7)^ Numerous studies involving clinical patients,^(8)^ hospitalized patients,^(9)^ cirrhotic patients,^(10)^ stroke victims,^(11)^ surgical patients,^(12)^ cancer^(13)^ and kidney patients^(14)^ have been conducted with APMT; however, there are few studies evaluating severe cardiac patients.^(15)^ Therefore, there is no established cutoff point for all populations.^(16)^

Body composition abnormalities are more difficult to clearly characterize in the intensive care environment; therefore, a strategy that combines different tools may be more appropriate. The present study aimed to verify the relationship between the adductor pollicis muscle thickness test and SGA and to correlate it with other anthropometric methods.

## METHODS

This cross-sectional observational study evaluated patients admitted to the ICU of a reference cardiology hospital in the state of Rio Grande do Sul, Brazil. The study was conducted in accordance with the principles of the current revision of the Declaration of Helsinki, the most recent version of the Good Clinical Practice Guidelines and Resolution 466/12. It was approved by the Research Ethics Committee of the *Fundação Universitária de Cardiologia* under number UP 4957/14.

All patients of both genders admitted to the ICU of the institution who were more than 18 years old, who agreed to undergo the assessment and who signed the Terms of Free and Informed Consent (TFIC) were included in the study. In case of a patient's inability to respond to and sign the TFIC, this agreement was solicited from the family or guardian. Patients with diseases that could exert a negative influence on muscle tropism (except malnutrition), chronic degenerative or inflammatory disorders, peripheral neuropathy, cancer, acquired immune deficiency syndrome, inflammatory bowel disease, neurological and motor disorders, with amputation of any limb, anasarca and those whose data collection was not possible within the first 48 hours of admission were excluded.

The nutritional status assessment and anthropometric measurements were performed by the nutritionist responsible for the research in the ICU. Data were collected for SGA^(5)^ and APMT measurements.^(7)^ Laboratory parameters, length of stay and vital signs were collected later from the electronic medical records and nursing spreadsheet. To evaluate the SGA, the patient or family member answered questions relating to weight loss, eating habits, gastrointestinal symptoms, functional capacity, disease and physical examination. The patients were then classified by SGA into well nourished (A), moderately malnourished (B), and severely malnourished (C).^(5)^ For statistical analysis, these data were transformed into dichotomous variables: no nutritional risk (nourished) and at nutritional risk (moderately malnourished and severely malnourished).

The weight and height measurements were either reported or estimated, as the ICU did not have an available bed scale or stadiometer, and patients were not clinically able to walk. Body mass index (BMI) was calculated and classified according to age. For seniors ≥ 60 years, Lipschitz's^(17)^ criteria for BMI were used, and for those between 18 and 59 years of age, the 1998 World Health Organization (WHO) criteria were used.^(18)^

Calf circumference (CC) was obtained with an inelastic and flexible tape measure with an accuracy of 1 mm and was measured at the midpoint of the right leg, flexed at 90°. Male and female patients with a circumference of < 31cm were considered to be at nutritional risk.^(19)^ The APMT measurement was performed with the patient seated, with arms flexed at approximately 90°, using a Cescorf^®^ brand skinfold caliper (Porto Alegre, RS, Brazil), exerting a continuous pressure of 10g/mm^2^ to pinch the adductor muscle in an imaginary triangle vertex formed by the extension of the thumb and forefinger. The procedure was performed on both hands three times, and the average value was used as the APMT measurement.^(7)^ As there is no cut-off point defined for this population, an article on valve surgery patients was used, which reported that an APMT thickness of < 6.5mm was associated with infectious complications.^(15)^

The collected data were entered into a database and evaluated using version 22 of the Statistical Package for the Social Sciences (SPSS), version 2.2, with a significance level of 0.05. The Chi Square and Fisher tests and, when necessary, the Mann-Whitney and Pearson correlation tests, were used for statistical analysis. The receiver operating characteristic (ROC) curve was used to evaluate the accuracy of the APMT evaluation for the dominant hand. All patients included in the present study were right-handed.

## RESULTS

A total of 86 patients were evaluated between August and November 2014. One patient was excluded due to a positive diagnosis for acquired immunodeficiency syndrome (AIDS) and two because it was not possible to evaluate the APMT, for a total of 83 patients included in the study. The study population consisted of 52 men with an average age of 68.6 ± 12.5 years, with a minimum of 35 years and a maximum of 98 years. Other anthropometric and laboratory data are shown in table 1.

**Table 1:** General characteristics of patients

	N	Means and standard deviations
Age (years)	83	68.66 ± 12.54
Weight (kg)	83	73.38 ± 15.96
Height (m)	83	1.66 ± 0.08
BMI (kg/m^2^)	83	26.56 ± 4.96
Calf circumference - (cm)	83	34.72 ± 4.14
APMT.R (mm)	83	8.03 ± 2.98
APMT.E (mm)	83	7.3 ± 2.71
Temperature (°C)	82	35.79 ± 0.93
Mean arterial pressure (mmHg)	81	93.4 ± 29.45
Heart rate (bpm)	82	69.46 ± 21.02
Respiratory rate (irpm)	82	19.89 ± 5.09
Sodium (mEq/L)	75	140.15 ± 4.05
Potassium (mEq/L)	77	4.44 ± 0.59
Hematocrit (%)	79	38.57 ± 6.91
Leukocytes (mg/dL)	79	9.61 ± 3.69

BMI - body mass index; APMT.R - adductor pollicis muscle thickness - right hand; APMT.L -adductor pollicis muscle thickness - left hand.

The most frequent cause of hospitalization was acute myocardial infarction (34.9%), followed by angina (24.1%), complete atrioventricular block (10.8%), pacemaker exchange (6%), aortic aneurysm (4.8%), congestive heart failure (CHF) (2.4%), stroke (1.2%) and other cardiac comorbidities (11.6%).

The following pathologies were among the most common pathologies observed: systemic arterial hypertension (SAH) in 63.9% of patients, followed by *diabetes mellitus* (DM) in 28.9%, coronary artery disease (CAD) in 25.3%, CHF in 12%, stroke in 12% and other cardiac pathologies in 27.7%.

The study population was mostly composed of elderly patients (61; 73.5%). Most of the elderly patients were classified as overweight (32.5%); 22.9% were normal weight, and 18.1% were malnourished. Patients aged between 18 and 60 were mostly classified as overweight (21.6% of total sample), and 4.8% of the total sample were between 18 and 60 years old and eutrophic.

According to the SGA, 62.7% of patients were well nourished, 20.5% moderately malnourished and 16.9% severely malnourished. Upon combining the moderately malnourished and severely malnourished groups, 54.8% of women were at higher risk of malnutrition (p = 0.011); the same result occurred with those who had an APMT score of < 6.5 (Table 2). As expected, according to both SGA and APMT, older patients were at higher nutritional risk. Mortality was also higher in the SGA nutritional risk group, even though this finding was not statistically significant.

**Table 2:** Subjective global assessment and adductor pollicis muscle thickness (dominant hand)

Variables	SGA			APMT		
	No nutritional risk	With nutritional risk	p value	No nutritional risk > 6.5mm*	With nutritional risk < 6.5mm*	p value
	N = 52	N = 31		N = 55	N = 28	
Age (years)						
> 60	34 (53.1)	30 (45.9)		38 (59.4)	26 (40.5)	
18 - 59	18 (94.7)	01 (5.3)	0.003	17 (89.5)	02 (10.5)	0.03
Gender						
Female	14 (26.9)	17 (54.8)		13 (23.6)	18 (64.3)	0.001
Male	38 (73.1)	14 (45.2)	0.011	42 (76.4)	10 (35.7)	
BMI (Lipschitz^(17)^; WHO^(18)^)						
Malnourished	1 (1.9)	14(15.2)		4 (7.3)	11 (39.3)	
Eutrophic	13(25.2)	10 (32.3)	0.001†	14 (25.5)	9 (32.1)	0.001†
Overweight	38 (73.1)	7 (22.6)		37 (67.3)	8 (28.6)	
CAD	14 (29.8)	7 (22.6)	0.483	18 (36)	3 (10.7)	0.016
SAH	32 (69.6)	21 (67.7)	0.865	31 (63.3)	22 (78.6)	0.163
DM	13 (28.3)	11 (35.5)	0.502	12 (24.5)	12 (42.9)	0.094
Dyslipidemia	14 (30.4)	5 (16.1)	0.153	12 (24.5)	7 (25)	0.96
CHF	1 (2.2)	9 (29)	0.001	1 (2)	9 (32.1)	0.001
Stroke	4 (58.7)	6 (19.4)	0.172	5 (10.2)	5 (17.9)	0.337
Other pathologies	14 (30.4)	9 (29)	0.895	14 (28.6)	9 (32.1)	0.742
Death	1 (25)	3 (75)	0.127	2 (3.8)	2 (7.1)	0.519
Time in ICU**	3 (2 - 5.75)	3.5 (2 - 6)	0.845	4 (3 - 6)	3 (2 - 4.77)	0.183
Hospitalization time**	7 (5 - 9.8)	9 (4 - 14)	0.236	8 (6 - 13)	7 (4 - 10)	0.411

SGA - subjective global assessment; APMT - adductor pollicis muscle thickness; BMI - body mass index; DAC - coronary artery disease; SAH - systemic arterial hypertension; DM - diabetes mellitus; CHF - congestive heart failure; ICU - intensive care unit.

* Cutoff point suggested for this population^(15)^;

† Pearson chi square test;

** median and interquartile range, Mann-Whitney U-test. The results expressed as number (percentage).

CHF showed a higher nutritional risk according to the SGA and the APMT - 9 of 10 CHF patients were at nutritional risk (p = 0.001) (Table 2).

The length of ICU hospitalization had an asymmetric distribution, hence the need for a median (Table 2).

**Figure 1:**
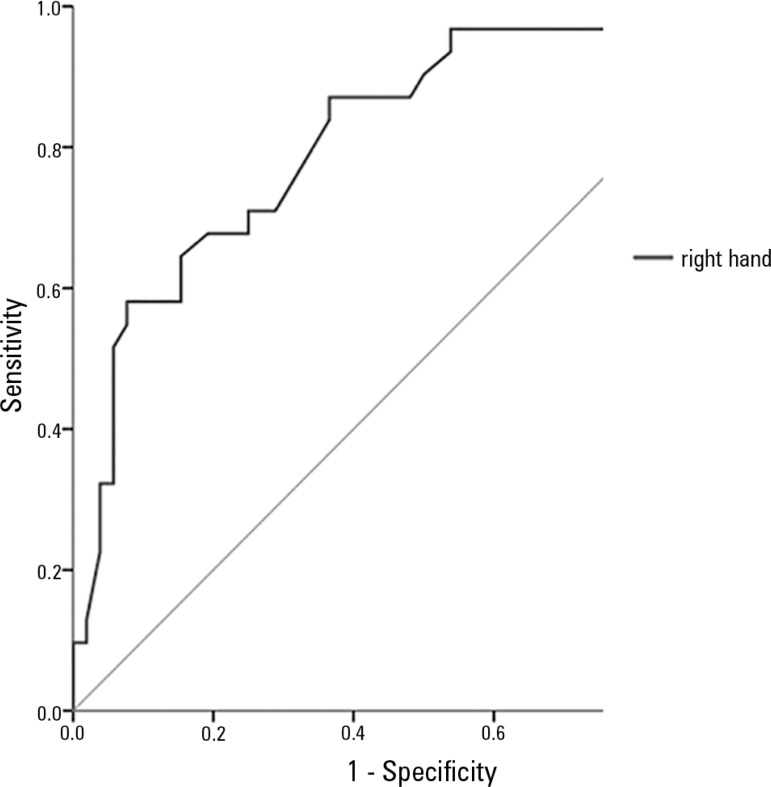
ROC curve for right-hand adductor pollicis muscle thickness in relation to nutritional risk evaluated by subjective global assessment (area under the curve relative to a right-hand measurement of 0.82; 95% confidence interval from 0.73 to 0.91).

In assessing the ROC curve of the right-hand APMT.R correlation with SGA, the area under the curve was 0.822 (Figure 1), which demonstrates the accuracy of the APMT test.

**Figure 2:**
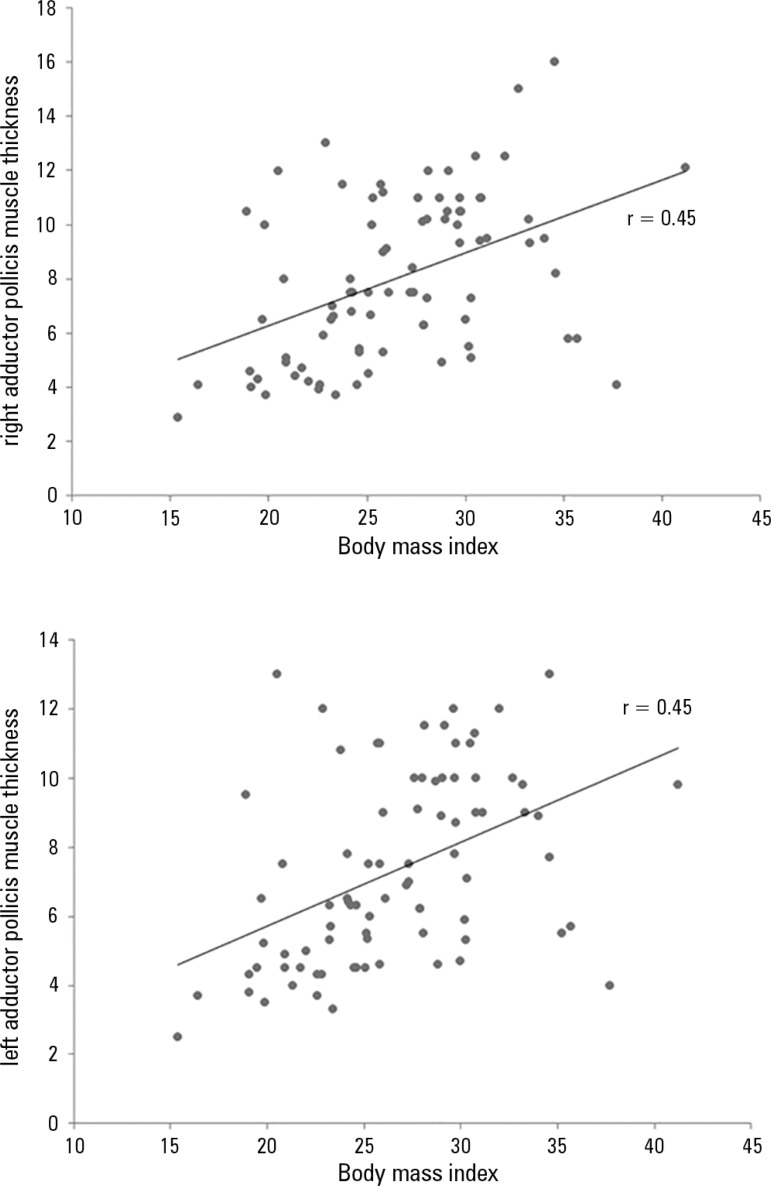
Relationship between adductor pollicis muscle thickness values of both hands and body mass index. p < 0.05 in all correlations; r = 0.45.

**Figure 3:**
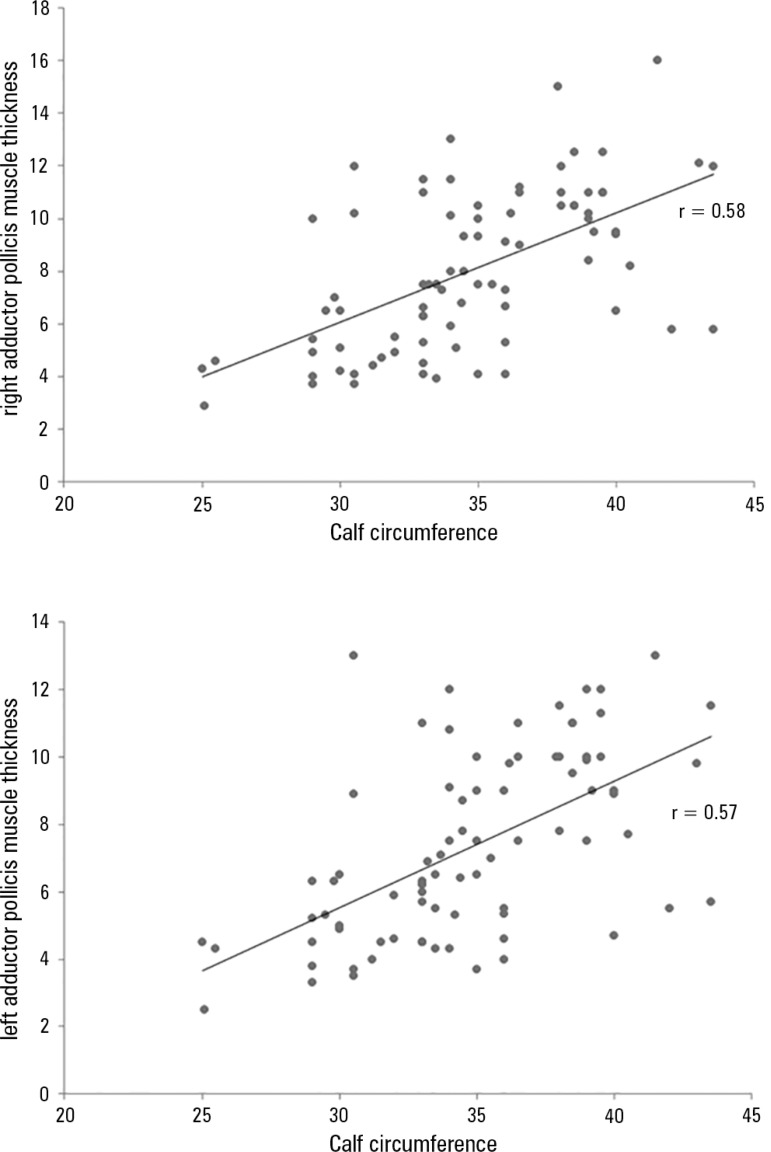
Correlation of adductor pollicis muscle thickness for both hands with anthropometric calf circumference measurements. p < 0.05 in all correlations; r = 0.58.

In correlating the APMT of the right hand with BMI (Figure 2), BMI and APMT.L (r = 0.44; p < 0.001) and BMI and APMT.R (r = 0.45; p < 0.001), the association was weak but significant, with a positive correlation. Figure 3 shows that there were also correlations between APMT.L and CC (r = 0.57; p < 0.001) and BMI and APMT.D (r = 0.58; p < 0.001).

## DISCUSSION

In the present study, the population mostly consisted of elderly individuals (73.5%), with an average age of 68.6 years. As the elderly population is more prone to hospital malnutrition,^(2)^ the importance of implementing screening is evident, along with specific nutritional assessment for this population. These measures could help in early diagnosis and proper nutritional intervention^(4)^ given that, globally, the majority of severely ill patients do not receive proper nutrition during hospitalization in the ICU.^(20)^

Correct nutritional assessment is a challenge. More research is being conducted to identify the best tool to be used, as they vary in terms of diagnosis, overestimating or underestimating nutritional risk.^(6,21,22)^ Upon analysis of the methods used in the present study, the tools showed small diagnostic differences, but both SGA and APMT showed relationships of accuracy, with areas under the curve (AUC) of 0.822 for both sensitivity and specificity. These data corroborate a cross-sectional study of patients eligible for surgical procedures, which evaluated SGA, APMT and other anthropometric and biochemical measures. In that study, APMT proved to be a reliable method for assessing the nutritional status of surgical patients when the results of this method were compared with the gold standard, SGA (area under the curve of 0.93).^(12)^

In research with valve surgery patients, APMT showed an association with postoperative complications, using sensitivity and specificity assessment with an area under the curve of 0.624, characterizing the presence of septic complications in APMT < 6.5mm.^(15)^ In our findings, values of < 6.5mm were related to greater nutritional risk according to the SGA.

When we evaluated average APMT among our patients, from 7.3 ± 2.71mm to 8.03 ± 2.98mm (left hand and right, respectively), lower values were found compared to other studies. In a study investigating APMT in healthy people with an average age of 44.9 ± 18.5 years, the averages for men and women were 26.1 ± 4.4mm and 19.8 ± 3.3mm, respectively.^(23)^ In surgical patients, the average APMT for the right hand was 12.64 ± 3.19 mm; for the left hand, the average APMT was 12.23 ± 2.9mm.^(12)^ When studying valve surgery patients, Andrade et al. found an average APMT value of 11.5mm.^(15)^

We believe that the low APMT value encountered in our results is because most of the studied population consisted of individuals over 60 years old (73%) and therefore with decreased muscle mass.

When correlated with BMI and CC, APMT.R showed a positive correlation, which is in line with the findings of Bragagnolo et al., according to whom APMT correlated with all of the classic anthropometric measurements, demonstrating the efficiency of the test.^(12)^

The SGA results were similar to those of a prospective cohort study with elderly patients with an average age of 74.2 years. The SGA in that study identified 21% as moderately malnourished at ICU admission and was associated with increased length of hospital stay, a lower propensity to be discharged, a greater need for palliative care and death at discharge (all p values < 0.05). These results did not correspond with the present study, which did not observe a relationship with hospitalization time.^(21)^

Among the investigated conditions, the disorder that showed significance with nutritional risk (SGA) and muscle loss by APMT was CHF, affecting mainly the elderly. These two nutritional assessment methods corroborate physiological changes caused by this syndrome, changes in cardiac output and impaired systemic circulation, leading to dyspnea, edema, fatigue^(24)^ and loss of muscle mass, regardless of total body mass.^(25)^

The limitations of the present study involved the difficulty in collecting anthropometric data in the first 48 hours of admission, as most patients were unable to have their weight and height measured, which led to the use of data reported by the patient or family.

## CONCLUSION

All nutritional assessment methods (subjective global assessment, body mass index, calf circumference and adductor pollicis muscle thickness) showed differences in their results; however, they were efficient and positively correlated with the diagnosis of nutritional risk. Subjective global assessment was the most reliable method of classifying nutritional risk.

Adductor pollicis muscle thickness proved to be a good method for evaluating nutritional risk, as it was accurate when compared to the gold standard, which is subjective global assessment. However, studies with a larger population that are able to establish a cutoff and to demonstrate a relationship with outcomes and complications in the cardiac intensive care unit are required.
